# Supplementary material for: Perspectives from designated family caregivers of critically ill adult patients during the COVID-19 pandemic: A qualitative interview study
Source: PLoS One. 2022 Sep 27;17(9):e0275310. doi: 10.1371/journal.pone.0275310 (PMC9514636; doi:10.1371/journal.pone.0275310)
Supplement: S1 Table — (DOCX) [file pone.0275310.s001.docx]

**Supplemental Table 1. Consolidated criteria for reporting qualitative studies (COREQ)**

| **No. Item** | **Guide Questions or Description** | **Reported on Page #** |
| --- | --- | --- |
| **DOMAIN 1: RESEARCH TEAM AND REFLEXIVITY** | | |
| *Personal Characteristics* |  |  |
| 1. Interviewer/facilitator | Which author/s conducted the interview or focus group? | Results |
| 2. Credentials | What were the researcher’s credentials? | Methods |
| 3. Occupation | What was their occupation at the time of the study? | Methods |
| 4. Gender | Was the researcher male or female? | N/A |
| 5. Experience and training | What experience or training did the researcher have? | Methods |
| *Relationship with participants* |  |  |
| 6. Relationship established | Was a relationship established prior to study commencement? | N/A |
| 7. Participant knowledge of the interviewer | What did the participants know about the researcher? | N/A |
| 8. Interviewer characteristics | What characteristics were reported about the inter viewer/facilitator? | Methods |
| **DOMAIN 2: STUDY DESIGN** |  |  |
| *Theoretical framework* |  |  |
| 9. Methodological orientation and Theory | What methodological orientation was stated to underpin the study? | Methods |
| *Participant selection* |  |  |
| 10. Sampling | How were participants selected? e | Methods |
| 11. Method of approach | How were participants approached? | Methods |
| 12. Sample size | How many participants were in the study? | Results |
| 13. Nonparticipation | How many people refused to participate or dropped out? Reasons? | Methods |
| *Setting* |  |  |
| 14. Setting of data collection | Where was the data collected? | Methods |
| 15. Presence of non-participants | Was anyone else present besides the participants and researchers? | Results |
| 16. Description of sample | What are the important characteristics of the sample? | Results |
| *Data collection* |  |  |
| 17. Interview guide | Were questions, prompts, guides provided by the authors? Was it pilot tested? | Methods |
| 18. Repeat interviews | Were repeat inter views carried out? If yes, how many? | N/A |
| 19. Audio/visual recording | Did the research use audio or visual recording to collect the data? | Methods |
| 20. Field notes | Were ﬁeld notes made during and/or after the interview or focus group? | Methods |
| 21. Duration | What was the duration of the inter views or focus group? | Methods |
| 22. Data saturation | Was data saturation discussed? | Methods |
| 23. Transcripts returned | Were transcripts returned to participants for comment and/or correction? | N/A |
| **DOMAIN 3: ANALYSIS AND FINDINGS** | |  |
| *Data analysis* |  |  |
| 24. Number of data coders | How many data coders coded the data? | Methods |
| 25. Description of the coding tree | Did authors provide a description of the coding tree? | N/A |
| 26. Derivation of themes | Were themes identiﬁed in advance or derived from the data? | Methods |
| 27. Software | What software, if applicable, was used to manage the data? | NVivo |
| 28. Participant checking | Did participants provide feedback on the ﬁndings? | Strengths and limitations |
| *Reporting* |  |  |
| 29. Quotations presented | Were participant quotations presented to illustrate the themes/ﬁndings? Was each quotation identiﬁed? | Results |
| 30. Data and ﬁndings consistent | Was there consistency between the data presented and the ﬁndings? | Relationship to existing knowledge |
| 31. Clarity of major themes | Were major themes clearly presented in the ﬁndings? | Results |
| 32. Clarity of minor themes | Is there a description of diverse cases or discussion of minor themes? | Discussion |
