# Supplementary material for: Perspectives from designated family caregivers of critically ill adult patients during the COVID-19 pandemic: A qualitative interview study
Source: PLoS One. 2022 Sep 27;17(9):e0275310. doi: 10.1371/journal.pone.0275310 (PMC9514636; doi:10.1371/journal.pone.0275310)
Supplement: S2 Table — (DOCX) [file pone.0275310.s002.docx]

**Supplemental Table 2.** Interview Guide

**Perspectives on caring for the critically ill**

(follow-up interviews for the ACTIVATE Trial)

Semi-structured interview guide

**AT START**

Name

M / F

COVID?

**AT END**

Relationship

Primary Y / N

Live with?

1. **Introduction**

****If you find the interview is running long, emphasize probing over asking many questions****

**Interviewer Introduction:** My name is Stephana, and I am PhD Candidate in the Department of Critical Care Medicine. My PhD research focuses on patient and family-centered critical care. I am examining the health effects of caregiving for a critically ill patients, to describe the caregiving skills and experiences that promote better patient and caregiver outcomes.

**Participant Introduction:** (Participant introduce him/herself [name, where from]).

**Length:** The length of this interview will be about 30 to 45 minutes

**Primary goal:** The primary goal of this interview is to see the things the way you see them. My goal is for us to engage in a conversation, with a focus on your experience, your opinions, and what you think or feel about the topics. We will focus on three areas of interest that will help us to better understand the overall caregiving experience.

**Experience:** The first area is your feelings about the general caregiving experience and its impact on your life and your relationships with others. I will ask you to think about the challenges and needs you have faced as a family caregiver for an ICU patient.

**Environment:** The second area is about the environments that you have been a caregiver in. I would like to know about how your experience as a caregiver has changed depending on being in the ICU and at home after your family member was discharged. I will ask about how your needs and perceptions changed over time following ICU discharge.

**Coping:** The third and final area is about different coping methods that you have used in your experience as a caregiver. I will ask about the strategies or skills that you have used to cope with the challenges and needs you have faced as a family caregiver. I am interested to know what has/has not worked, and the lessons you have learned.

**Recording:** As a part of the ACTIVATE Trial, we are trying to learn about what it’s like to be a caregiver for a critically ill patient. We value your perspectives on how this can be improved. I hope you will permit me to record our conversation today. Recording the interview allows me to be more attentive to our conversation and it also allows me to create a written record of the exact conversation, which I can return to you to be certain that I have understood the conversation correctly. When we are finished this interview, I will assign a participant ID to your transcript and all your identifying information will be removed. Your responses will be kept confidential. Your participation in this interview is voluntary and you can skip any questions that you are not comfortable answering. You can request to end the interview at any time.

1. ***Verbal consent***

Now I would like to ask for your verbal consent. Would you like to participate in this interview?

**O** Verbal consent was obtained from the study participant

**O** Verbal consent was NOT obtained from the study participant

Do you permit me to record our interview?

**O** The interviewee permitted recording

**O** The interviewee did NOT permit recording

Do you have any questions about confidentiality or about the ACTIVATE Trial in general?

**O** The interview proceeded further

**O** The interview did NOT proceed further

1. ***Background information***

*This is a conversational start in order to put the participant at their ease. We are trying to get a sense of their overall caregiver literacy so that we can set their caregiving behaviors within a broader context.*

First, I would like to know a bit of background information on your role as being a caregiver. Please think about your current typical day as being a caregiver. What does this day look like for you right now?

[PROBE:

What are the good things about being a caregiver?

What are some of the things that you enjoy as being a caregiver?

What are some of the barriers that you face as being a caregiver?]

**It sounds like your role as being a caregiver is _______________________.**

Now I would like to know whether or not your loved one developed delirium in the ICU. Did your loved one develop delirium in the ICU?

[PROBE:

Did your loved one have symptoms of delirium when they were in the ICU?

Was your loved one delirious when they were in the ICU?]

*If the participant identifies that their patient had delirium, ask the next two questions.*

How did you participate in detecting that your loved one had developed delirium?

[PROBE:

How did you know that your loved one had developed delirium?

How did you know that your loved one was showing signs or symptoms of delirium?]

Did your caregiving role become more challenging after your loved one developed delirium?

[PROBE:

Was it harder to be a caregiver for your loved one after their delirium diagnosis?]

Now I would like to know whether or not your loved one was diagnosed with the coronavirus when they were in the ICU. Was your loved one diagnosed with COVID in the ICU?

[PROBE:

Did your loved one have symptoms of COVID when they were in the ICU?]

*NB- no patients had COVID when they were in the ICU.*

Was your loved one diagnosed with COVID after discharge from the ICU?

[PROBE:

Did your loved one have symptoms of COVID after they were discharged from the ICU?]

Was your caregiving role more challenging after your loved one was diagnosed with COVID?

[PROBE:

Was it harder to be a caregiver for your loved one after their COVID diagnosis?]

1. ***What matters to you?***

*We want to encourage a meaningful conversation with the participant. By asking what really matters, we can build trust, develop empathy, and understand the families. This simple question aims to reach the essence of person-centered care in a simple way. Ultimately, starting the interview with this question will improve the humanistic quality of the interview and the person-centered care that we may provide.*

Before we begin with the three main areas of experience, environment, and coping that I described earlier, I first would like to know, what matters to you today?

[PROBE:

Is there anything specific that you would like us to talk about today?]

****Please indicate below how the participant appears****

****This will aid in how the statements are analyzed regarding reflexivity and dissemination****

**O** The participant appears open to conversation and satisfied to start (positive)

**O** The participant appears closed to conversation and dissatisfied to start (negative)

1. ***Caregiving experience***

*We can transition from S4 to S5 based on the response and the tone to S4. The participant will be most likely to talk about what is currently on their mind. So, if the participant appears open to conversation, we can ask about what the most beneficial things have been (i.e., a positive experience). Accordingly, if the participant appears closed to conversation, we can ask what the most difficult things have been (i.e., a negative experience).*

I would now like to begin with the first area of interest that is the overall caregiving experience. Please can you describe to me your thoughts and perceptions about the general experience of being a caregiver for your critically ill loved one?

[PROBES:

Overall have you had a positive or negative experience?

Open: What do you see as the benefit of being a caregiver?

Closed: What has been the most difficult thing you have had to help with?]

1. ***Caregiving environment***

*We can transition from S5 to S6 based on the environment primarily described in S5. If the participant mainly talks about the ICU in S5, transition to S6 by asking further questions related to caregiving in the ICU. If the participant mainly talks about being at home in S5, then transition to S6 by asking further questions related to caregiving at home. Regardless, let the participant speak about both ICU and home.*

Now I would like to focus on the second area of interest that is the environment where you have been a caregiver for your critically ill loved one. Please think back to what it was like to be a caregiver in the ICU. Can you describe a typical day?

[PROBES:

How much time on average did you spend caregiving in the ICU?

Would you consider yourself the primary family caregiver in the ICU?

What were some of the challenges and needs that you have faced in the ICU?

[PROBES:

What bothered you about being in the ICU?

How did your loved one’s healthcare team help you in the ICU?

How could your loved one’s healthcare team have helped you more in the ICU?]

Now please think about what it has been like to be a caregiver at home, after your loved one was discharged from the ICU. Can you describe what a typical day looked like then?

[PROBES:

How has the transition from ICU to home been for you as a caregiver?

Did your overall caregiving experience shift to be more positive or more negative?

How much time on average do you spend caregiving at home?

Would you consider yourself the primary caregiver at home?]

What were some of the challenges and needs that you have faced at home?

[PROBES:

What bothers you about caregiving at home?

How did you loved one’s health care team prepare you to be a caregiver at home?

How could your loved one’s health care team have prepared you more?]

1. ***Caregiver coping***

*It may feel more organic to discuss coping strategies that the caregiver has used in S6 when discussing the challenges and needs they have faced. For example, in S6 if the caregiver were to identify a specific challenge or need that they described deeply, you should then ask them if they would consider this one of the most significant challenges or needs that they have faced, and then segue in to S7. You may need to do this both for the ICU and the home environment. Alternatively, S6 and S7 can be separate sections.*

Now on to our final area of interest that is coping strategies and skills that you have used as a caregiver for your critically ill loved one. Please think of the most significant challenges and needs that you have faced, and the ways that you have coped with these significant challenges and needs. Can you tell me about how you have coped?

[PROBES:

*Assess behavioral coping through the following:*

How do you keep track of things?

What organization strategies do you have for being a caregiver?

What “things” do you use the most to help with being a caregiver?

Is there any “thing” that is absolutely necessary for your role as a caregiver?

*Assess cognitive coping through the following:*

How do you deal with the stress of being a caregiver?

Did you learn to deal with the stress yourself or with help?

Self: What past experiences did you draw on to deal with the stress?

Help: What sources did you/did you not use? How did you decide?

*Assess avoidance coping through the following:*

Would you identify yourself as a caregiver to others?

How important is being a caregiver to you?

Have there been times when you did not use recommended strategies?]

*If the participant has implemented consistent coping strategies, ask the next question.*

This next question will help us to produce actionable outcomes from our study. Overall, would you say that the coping strategies you have used have worked or not worked to help you manage the challenges and needs that you faced being a caregiver for your loved one?

[PROBES:

*If they worked…*

What strategy was the most helpful?

*If they did not work…*

What strategy was the least helpful?]

1. ***Open-ended comments or questions***

*We want to end the interview on a substantive note. To do this, we will return to short and easy questions. As well, we saved these questions for the end because at this point we (the interviewer) are no longer a stranger but a pleasant, non-argumentative professional who seems genuinely interested in the participant’s opinions. So at this stage, we can introduce potentially sensitive questions. Please remind the participant of our confidentiality as needed.*

**These are all of the questions that I have for you.**

Do you have any questions for me about the ACTIVATE trial or this interview?

Is there anything you would like to add in light of what we have been talking about?

Is there anything you thought I would ask but did not, or anything else that you think is important for us to consider?

**Thank you for your participation in this interview. On behalf of the entire ACTIVATE Trial Team, we appreciate that you took the time to talk to us.**

*****REMEMBER TO COMPLETE (AT END) DEMOGRAPHICS IF NOT EVIDENT FROM RESPONSES*****
