# Supplementary material for: Perspectives from designated family caregivers of critically ill adult patients during the COVID-19 pandemic: A qualitative interview study
Source: PLoS One. 2022 Sep 27;17(9):e0275310. doi: 10.1371/journal.pone.0275310 (PMC9514636; doi:10.1371/journal.pone.0275310)
Supplement: Attachment — Submitted filename: PONE-D-21-35872.pdf [file pone.0275310.s003.pdf]

PLOS ONE

Perspectives from Designated Family Caregivers of Critically Ill Adult Patients During the COVID-19 Pandemic: A Qualitative Interview Study

--Manuscript Draft--

**Manuscript Number:**	PONE-D-21-35872
**Article Type:**	Research Article
**Full Title:**	Perspectives from Designated Family Caregivers of Critically Ill Adult Patients During the COVID-19 Pandemic: A Qualitative Interview Study
**Short Title:**	Perspectives from Designated Family Caregivers of Critically Ill Adult Patients During the COVID-19 Pandemic
**Corresponding Author:**	Kirsten M. Fiest<br>University of Calgary<br>Calgary, CANADA
**Keywords:**	Critical Care; Family; Interviews; COVID-19; Restricted Visitation
**Abstract:**	Background: Family visitation in intensive care units (ICU) has been impacted by the severe acute respiratory syndrome coronavirus 2 (COVID-19) pandemic. While studies report on perceptions of families completely restricted from ICUs, little is known about the burden experienced by designated family caregivers allowed to visit their critically ill loved one. This study sought the perspectives of family caregivers of critically ill patients on the impact of one-person designated visitor policies mandated in ICUs during the COVID-19 pandemic.<br>Methods: Throughout the study period a restricted visitation policy was mandated capturing the first and second wave of the pandemic that allowed one designated family caregiver (i.e., spouses or adult children) per patient to visit the ICU. Designated family caregivers of critically ill patients admitted to ICU September 2020 to November 2020 took part in individual 60-minute, semi-structured interviews at 6-months after discharge from the index ICU admission. Themes from family interviews were summarized with representative quotations. Need date, precise period<br>Results: Key themes identified following thematic analysis from six participants included: one visitor rule, patient advocate role, information needs, emotional distress, strategies for coping with challenges, practicing empathy, and appreciation of growth.<br>Conclusion: Designated family caregivers of critically ill patients admitted to ICU during the COVID-19 pandemic perceived a complex and highly stressful experience. Support from ICU family liaisons and psychologists may help ameliorate the impact.
**Order of Authors:**	Stephana J. Moss
	Karla D. Krewulak
	Henry T. Stelfox
	Scott B. Patten
	Christopher J Doig
	Jeanna Parsons Leigh
	Kirsten M. Fiest
**Additional Information:**	
**Question**	**Response**
**Financial Disclosure**<br><br>Enter a financial disclosure statement that describes the sources of funding for the work included in this submission. Review the submission guidelines for detailed requirements. View published research	SJM was supported by a Canadian Institutes of Health Research Doctoral Research Award. The funder had no role in study design, data collection and analysis, decision to publish, or preparation of the manuscript; no financial relationships with any organizations that might have an interest in the submitted work in the previous three years; no other relationships or activities that could have influenced the submitted work.

Powered by Editorial Manager® and ProduXion Manager® from Aries Systems Corporation

articles from *PLOS ONE* for specific examples.<br><br>This statement is required for submission and **will appear in the published article** if the submission is accepted. Please make sure it is accurate.<br><br>**Unfunded studies**<br>Enter: *The author(s) received no specific funding for this work.*<br><br>**Funded studies**<br>Enter a statement with the following details:<br>• Initials of the authors who received each award<br>• Grant numbers awarded to each author<br>• The full name of each funder<br>• URL of each funder website<br>• Did the sponsors or funders play any role in the study design, data collection and analysis, decision to publish, or preparation of the manuscript?<br>• **NO** - Include this sentence at the end of your statement: *The funders had no role in study design, data collection and analysis, decision to publish, or preparation of the manuscript.*<br>• **YES** - Specify the role(s) played.<br><br>* typeset	
**Competing Interests**<br><br>Use the instructions below to enter a competing interest statement for this submission. On behalf of all authors, disclose any competing interests that could be perceived to bias this work—acknowledging all financial support and any other relevant financial or non-financial competing interests.<br><br>This statement is required for submission and **will appear in the published article** if the submission is accepted. Please make sure it is accurate and that any funding sources listed in your Funding Information later in the submission form are also declared in your Financial Disclosure statement.	The authors have declared that no competing interests exist.

Powered by Editorial Manager® and ProduXion Manager® from Aries Systems Corporation

View published research articles from [*PLOS ONE*] for specific examples.<br><br>**NO authors have competing interests**<br><br>Enter: *The authors have declared that no competing interests exist.*<br><br>**Authors with competing interests**<br><br>Enter competing interest details beginning with this statement:<br><br>*I have read the journal's policy and the authors of this manuscript have the following competing interests: [insert competing interests here]*<br><br>* typeset	
**Ethics Statement**<br><br>Enter an ethics statement for this submission. This statement is required if the study involved:<br><br>• Human participants<br>• Human specimens or tissue<br>• Vertebrate animals or cephalopods<br>• Vertebrate embryos or tissues<br>• Field research<br><br>Write "N/A" if the submission does not require an ethics statement.<br><br>General guidance is provided below. Consult the submission guidelines for detailed instructions. **Make sure that all information entered here is included in the Methods section of the manuscript.**	The Conjoint Health Research Ethics Board at the University of Calgary approved this study (Ethics ID: REB19-1000). Informed and oral consent were sought for all participants of the study.

**Format for specific study types**<br><br>**Human Subject Research (involving human participants and/or tissue)**<br>• Give the name of the institutional review board or ethics committee that approved the study<br>• Include the approval number and/or a statement indicating approval of this research<br>• Indicate the form of consent obtained (written/oral) or the reason that consent was not obtained (e.g. the data were analyzed anonymously)<br><br>**Animal Research (involving vertebrate animals, embryos or tissues)**<br>• Provide the name of the Institutional Animal Care and Use Committee (IACUC) or other relevant ethics board that reviewed the study protocol, and indicate whether they approved this research or granted a formal waiver of ethical approval<br>• Include an approval number if one was obtained<br>• If the study involved *non-human primates*, add *additional details* about animal welfare and steps taken to ameliorate suffering<br>• If anesthesia, euthanasia, or any kind of animal sacrifice is part of the study, include briefly which substances and/or methods were applied<br><br>**Field Research**<br><br>Include the following details if this study involves the collection of plant, animal, or other materials from a natural setting:<br>• Field permit number<br>• Name of the institution or relevant body that granted permission	
**Data Availability**<br><br>Authors are required to make all data underlying the findings described fully available, without restriction, and from the time of publication. PLOS allows rare exceptions to address legal and ethical concerns. See the PLOS Data Policy and FAQ for detailed information.	No - some restrictions will apply

Powered by Editorial Manager® and ProduXion Manager® from Aries Systems Corporation

A Data Availability Statement describing where the data can be found is required at submission. Your answers to this question constitute the Data Availability Statement and **will be published in the article**, if accepted.<br><br>**Important:** Stating 'data available on request from the author' is not sufficient. If your data are only available upon request, select 'No' for the first question and explain your exceptional situation in the text box.<br><br>Do the authors confirm that all data underlying the findings described in their manuscript are fully available without restriction?	
**Describe where the data may be found in full sentences. If you are copying our sample text, replace any instances of XXX with the appropriate details.**<br><br>• If the data are **held or will be held in a public repository**, include URLs, accession numbers or DOIs. If this information will only be available after acceptance, indicate this by ticking the box below. For example: *All XXX files are available from the XXX database (accession number(s) XXX, XXX.*).<br>• If the data are all contained **within the manuscript and/or Supporting Information files**, enter the following: *All relevant data are within the manuscript and its Supporting Information files.*<br>• If neither of these applies but you are able to provide **details of access elsewhere**, with or without limitations, please do so. For example:<br><br>*Data cannot be shared publicly because of [XXX]. Data are available from the XXX Institutional Data Access / Ethics Committee (contact via XXX) for researchers who meet the criteria for access to confidential data.*<br><br>*The data underlying the results presented in the study are available from (include the name of the third party*	Data cannot be shared publicly because of patient confidentiality. Data may be available upon reasonable request from the University of Calgary research ethics board and Alberta Health Services research and innovation administration (contact via chreb@ucalgary.ca and research. administration@ahs.ca) for researchers who meet the criteria for access to confidential data.

and contact information or URL).<br>• This text is appropriate if the data are owned by a third party and authors do not have permission to share the data.<br><br>* typeset	
Additional data availability information:	

Manuscript Click here to access/download;Manuscript;Manuscript.docx

Manuscript

Perspectives from Designated Family Caregivers of Critically Ill Adult Patients During the COVID-19 Pandemic: A Qualitative Interview Study

Stephana J. Moss, MSc PhD[1,2,3,4]; Karla D. Krewulak, PhD[1,2]; Henry T. Stelfox, MD, PhD[1,2,3,5]; Scott B. Patten, MD PhD[1,3,456]; Christopher J. Doig, MD MSc[1,2,5]; Jeanna Parsons Leigh, PhD[2,6]; Kirsten M. Fiest, PhD[1,2,3,4,7]

[1]Department of Community Health Sciences, Cumming School of Medicine, University of Calgary, Calgary, AB, Canada
[2]Department of Critical Care Medicine, University of Calgary and Alberta Health Services, Calgary, AB, Canada
[3]O'Brien Institute for Public Health, University of Calgary, Calgary, AB, Canada
[4]Hotchkiss Brain Institute, University of Calgary, Calgary, AB, Canada
[5]Alberta Health Services, Edmonton, AB, Canada
[6]Faculty of Health, School of Health Administration, Dalhousie University, Halifax, NS, Canada
[7]Department of Psychiatry, Cumming School of Medicine, University of Calgary, Calgary, AB, Canada

Moss: stephana.moss@ucalgary.ca
Krewulak: kkrewula@ucalgary.ca

Manuscript

Stelfox: tstelfox@ucalgary.ca
Patten: patten@ucalgary.ca
Doig: cdoig@ucalgary.ca
Parsons Leigh: J.ParsonsLeigh@dal.ca
Fiest: kmfiest@ucalgary.ca (corresponding)

ABSTRACT

Background: Family visitation in intensive care units (ICU) has been impacted by the

Date ? Period?

severe acute respiratory syndrome coronavirus 2 (COVID-19) pandemic. While studies report on perceptions of families completely restricted from ICUs, little is known about the burden experienced by designated family caregivers allowed to visit their critically ill loved one. This study sought the perspectives of family caregivers of critically ill patients on the impact of one-person designated visitor policies mandated in ICUs during the COVID-19 pandemic.

Methods: Throughout the study period a restricted visitation policy was mandated capturing the first and second wave of the pandemic that allowed one designated family caregiver (i.e., spouses or adult children) per patient to visit the ICU. Designated family caregivers of critically ill patients admitted to ICU September 2020 to November 2020 took part in individual 60-minute, semi-structured interviews at 6-months after discharge from the index ICU admission. Themes from family interviews were summarized with representative quotations.

Manuscript

Results: Key themes identified following thematic analysis from six participants included: one visitor rule, patient advocate role, information needs, emotional distress, strategies for coping with challenges, practicing empathy, and appreciation of growth.

Conclusion: Designated family caregivers of critically ill patients admitted to ICU during the COVID-19 pandemic perceived a complex and highly stressful experience. Support from ICU family liaisons and psychologists may help ameliorate the impact.

Key Words: Critical Care; Family; Interviews; COVID-19; Restricted Visitation

INTRODUCTION

Critically ill patients admitted to the intensive care unit (ICU) are among the sickest patients in the healthcare system given their need for urgent treatment with life sustaining technologies [1]. Family caregivers of critically ill patients experience distress, as witnessing critical illness and intense ICU therapies can elicit feelings of helplessness [2]. Family caregivers frequently experience long-lasting, negative psychological consequences, including anxiety, depression, post-traumatic stress disorder, emotional distress, and sleep disturbances [3].

In response to the burden of critical illness for family caregivers of ICU patients, the Society of Critical Care Medicine Guideline for Patient and Family-Centred Care recommends regular visitation between family caregivers and ICU patients to improve outcomes (e.g., distress) [4, 5] and experiences (e.g., satisfaction) [6, 7] among ICU patients and their families. Most hospitals, including the intensive care units, enacted restricted visitation policies as part of infection control measures [8] to limit spread of the COVID-19 virus, reduce use of personal protective equipment, and to facilitate organizing care [9, 10]. Well-intentioned, restricted visitation policies may have unintended negative consequences on family caregivers, such as

grief over inadequate communication and sparse involvement in the provision of care [11, 12]. Designated family caregivers of critically ill patients admitted to ICUs that mandated one-person designated visitor policies faced additional challenges when having to deliver medical information to other family members that were restricted from visiting [13, 14].

Restricted visitation in the ICU during the COVID-19 pandemic may lead to long-term detriment [15]. Perspectives from designated family caregivers of critically ill patients are unknown. The objective of this study was to describe perspectives of designated family caregivers of critically ill patients on the impact of one-person designated visitor policies mandated in ICUs during the COVID-19 pandemic.

METHODS

Study Design

This qualitative study was conducted at Foothills Medical Centre ICU (Calgary, AB, Canada) between September 2020 to November 2020. A restricted visitation policy was mandated throughout the study period (March 2020 to May 2021); capturing the (entire) first and

second waves and (part of the) third wave of the pandemic that allowed one designated family caregiver per patient to visit. We used a qualitative descriptive approach [16] with data collected from semi-structured interviews with designated family caregivers (i.e., spouses or adult children designated to visit the ICU routinely) of critically ill patients in accordance with the Consolidated Criteria for Reporting Qualitative Research (COREQ) (S. Table 1) [17]. The Conjoint Health Research Ethics Board at the University of Calgary approved this study (Ethics ID: REB19-1000). Informed consent and oral consent were sought from all participants that agreed to be interviewed.

Selection and Description of Participants

We used a convenience sample, of designated family caregivers who participated in another study by our group and indicated interest in being contacted to participate in additional research projects. Family caregivers were adults (≥18 years), able to understand and communicate in English, and able to provide informed consent. We invited family caregivers using the contact information they provided (e-mail or telephone).

Semi-structured Interview Guide

Manuscript

A multidisciplinary research team (patient partner (B.S.), doctoral student (S.M.), research assistant (I.Y.), research associate (K.K), epidemiologist (K.F.), and qualitative research expert (J.P.L.)) created a draft semi-structured interview guide based on research experience and relevant literature [14, 18, 19]. For feedback and to ensure quality control, draft semi-structured interview guides were presented to a patient partner (M.A., a community member involved with our research team) and their family caregiver (J.A.), as well as a research coordinator (C.G.), all of whom had no prior involvement in the research study. A revised interview guide was then drafted, and pilot tested independently on three occasions in interviews with two critical care nurses (K.W., V.O.), and an intensivist (N.J.). The set interview guide was refined iteratively based on feedback from pilot interviews; no further edits were required after this point (S. Table 2).

Data Collection

How was selected the study population ? How many patients was asked to participate before reaching 6 people?

Demographic data on patients and family caregivers was collected upon enrollment in the larger RCT. Telephone interviews were conducted by S.M. who has experience planning and facilitating semi-structured interviews. Two days prior to each interview, participants (with e-mail access) were sent information about the interview objectives. Participant oral consent

was obtained by the research team prior to the start of each interview. All interviews were conducted within 60-minutes, audio recorded, transcribed verbatim, de-identified, and imported to NVivo-12 (QSR International, Melbourne, Australia) for data management.

Data Analysis

We analyzed demographic data by using descriptive statistics. All variables were categorical and reported as counts and proportions. Analysis of qualitative data was conducted concurrently and iteratively using a thematic synthesis approach published by Braun and Clarke [20]. We used a data-driven inductive approach to coding [21] that allowed our working knowledge of the topic [22] to guide the structure of interview discussions while permitting themes to emerge directly from the data [23]. The coding process included two coders (S.M., K.K.) who carefully read all transcripts before coding one-third of the data set to generate initial codes. Once the initial set of codes was developed, coders switched transcripts to ensure that all were coded in duplicate. The two coders searched for themes by collating codes across the data set and met biweekly for one month to refine themes and discuss progress. Two participants were provided with a copy of the final list of themes and sub-themes to review and comment on to ensure credibility, accuracy, and validity. We

formally compared themes across participants and compared interpretations across researchers in order to ensure analytic rigor.

RESULTS

Participants

Ten designated family caregivers participated in another study by our group from September 2020 to November 2020, of which eight (*n*=8, 80%) indicated interest in being contacted to participate in additional research projects through a telephone call (*n*=2, 25%) or an e-mail invitation (*n*=6, 75%) (Figure 1). Six (75%) family caregivers agreed to participate in the telephone interview. Interviews were conducted at an average of 6.3 months (standard deviation [SD] 2.3) post-ICU discharge.

To be in methodology. Need reference from original study.

Family caregivers were mostly female (*n*=4, 67%), of North American descent (*n*=4, 67%), and had completed some university/college, without receiving a degree (*n*=4, 67%). Half (*n*=3, 50%) of the participants were spouses of critically ill patients (Table 1). Some family caregivers (*n*=2, 33%) self-reported being diagnosed or treated for depression (prior to ICU

admission) and half (*n*=3, 50%) self-reported being diagnosed or treated for anxiety (prior to ICU admission). Critically ill patients were mostly male (*n*=4, 67%) with either some high school (*n*=2, 33%) or a Master's degree (*n*=2, 33%). No patient was diagnosed with the COVID-19 virus prior to or during their ICU stay.

These are data describing the patient, not the study population . To clarify

Table 1. Demographics of designated family caregiver participants and their critically ill loved one admitted to an intensive care unit during the COVID-19 pandemic

Demographic	Family Caregivers (N=6)	Critically Ill Patients (N=6)
Sex[1]		
Male	2 (33.3%)	4 (66.7%)
Female	4 (66.7%)	2 (33.3%)
Gender[2]		
Male	2 (33.3%)	4 (66.7%)
Female	4 (66.7%)	2 (33.3%)
Ethnic or cultural group[3]		
Other North American	4 (66.7%)	3 (50.0%)
First Nations	1 (16.7%)	0 (00.0%)
Eastern European	2 (33.3%)	2 (33.3%)
Western European	2 (33.3%)	1 (16.7%)
British Isles	1 (16.7%)	0 (00.0%)
Education[4]		
Some high school	0 (00.0%)	2 (33.3%)
High school graduate	0 (00.0%)	1 (16.7%)
Some university/college (no degree)	4 (66.7%)	0 (00.0%)
Bachelor's degree	2 (33.3%)	0 (00.0%)
Master's degree	0 (00.0%)	2 (33.3%)
Professional degree	0 (00.0%)	1 (16.7%)
Relationship to patient		
Spouse or Common-Law	3 (50.0%)	--
Adult Child	3 (50.0%)	--
Considered primary caregiver[5]		
Yes	6 (100.0%)	--
No	0 (00.0%)	--
Clinically relevant depression[6]		
Yes	2 (33.3%)	--
No	4 (66.7%)	--
Clinically relevant anxiety[6]		
Yes	3 (50.0%)	--
No	3 (50.0%)	--

Numbers are counts with percentages. Dashes indicate that the question was not asked.
[1]Recorded at birth
[2]Reported as gender identity
[3]Multiple selections per participant were allowed
[4]Highest degree received
[5]During patient stay in the ICU and after discharge from hospital
[6]Relating to direct medical treatment by a healthcare professional prior to ICU admission as self-reported by the participant

Family Caregiver Perceptions

Designated family caregivers of critically ill patients admitted to ICU during the COVID-19 pandemic perceived a complex and highly stressful experience. Support from ICU family liaisons and psychologists may help ameliorate the impact. Participants described their experiences with having to process their loved one's prognosis and treatment information, engage in shared decision making, and then relay information to family members who were not allowed to visit.

Comment from the study population or author?

Seven themes related to caring for a critically ill patient as the designated family caregiver during the COVID-19 pandemic were identified in the data: (1) one visitor rule; (2) patient advocate role; (3) information needs; (4) emotional distress; (5) strategies for coping with challenges; (6) practicing empathy; and (7) appreciation of growth (Table 2).

Confused, to be explain

Each point needs a small definition and how the classification was made

Table 2. Perspectives of designated family caregivers on caring for critically ill loved ones admitted to an intensive care unit during the COVID-19 pandemic

Themes	Quotes
**One Visitor Rule**	*At that time, one visitor was allowed that had to be the same visitor. So, our children were not allowed to visit, which was really hard on them all. Okay, we thought, we have to deal with this. (Spouse)*<br><br>*I just couldn't imagine him being there all by himself—I just had to be there, it was very important to me. (Son)*<br><br>*It was absolutely harder because of COVID. I felt like I had to be there all of the time because only one person was allowed, and I didn't ever want him to feel like he was alone. He didn't understand why the kids couldn't be there—that made it very hard, not being able to have that support. (Spouse)*
**Patient Advocate Role**	*I was there and I saw the lung exercises, so then I could quiz him later on. I asked, did you do it, do you remember how long you're supposed to do it? You're supposed to do it every hour. Are you doing it? Those people out there really care, and I want you to do your lung exercise. But what I know is that is that was just a phone call [if no visitors were allowed in the ICU]', I wouldn't know those specifics and I wouldn't be able to watch and be an advocate. (Spouse)*<br><br>*Even though I was the only one in there I was never asked to pipe up, to tell them about what he is really like, to advocate—I didn't know how you know, being alone. (Spouse)*<br><br>*The lessons learned is I wish I would have been more involved in rounding. I wish I would've been more invited. I know it's hard right now, given [COVID-19] restrictions, but if I was even just listening, I would have felt included. (Daughter)*
**Information Needs**	*I'm a very curious person and I like to know what's going on. So, I spent a long time asking questions whenever they were there. The staff was very good at giving us answers, but yet a lot of the time we were waiting—sitting and waiting or going back and forth and waiting for them to come to your patient. (Daughter)*<br><br>*I was afraid to ask questions. I felt isolated. Not a lot of information was given. I felt intimidated. (Spouse)*<br><br>*I had to give daily updates to everybody. I would wait for a report and then I would go out to call everybody, let them know how he was doing. So many calls—I had to make myself a pretty decent schedule. The only things I had time for were to come in [to the ICU], make all my calls, go back to the hotel, turn on TV for a few minutes and then go to bed. (Spouse)*
**Emotional Distress**	*It was an emotional and very difficult experience. I felt scared, left out, kind of anxious. (Daughter)*<br><br>*I spent many hours just sitting there wondering, you know, listening to machines, beeping, very loudly. I was on an emotional rollercoaster—I couldn't find solitude. (Spouse)*<br><br>*I think that you fool yourself into thinking that you're okay. I'm okay. I'm okay. I'm okay, I kept saying. You just are running on adrenaline, right. I didn't relax until he left ICU and I know it's the times, right. I had to tell myself that it was fine…initially anyway. (Spouse)*
**Strategies for Coping with Challenges**	*I knew my lifestyle. I had to eat better, you know, as far as getting some sleep at night. And so, sometimes I wouldn't come back a second time, and there was one time I remember I actually felt really guilty. (Son)*<br><br>*I'm very scheduled. So, I made myself a daily schedule. Mostly for my own mental health. (Son)*<br><br>*I made myself a decent schedule… day in and day out. That helped for all of us. (Spouse)*<br><br>*I wrote everything down. I would write it down, what the care team said for the entire day, and then at end of each day I would write everything in a second book that I left for my husband so that he could look back and find what he needed to know, even when I wasn't there. (Spouse)*
**Practicing Empathy**	Being able to talk to them [the ICU care team] provided a feeling of solidarity. They were going through a hard time. You know, you do take an interest in other people who are always there. The talking helped, like teamwork. (Son)<br><br>I would suggest a [virtual] peer group of people that have to deal with these issues, so we can exchange coping mechanisms and ideas, and show empathy for others, you know, that would be helpful. (Spouse)
**Appreciation of Growth**	*I mean, once you've been through these stressful, traumatic, draining situations, you look back and reflect and think, great, I can do tough things because I've dealt with a lot. (Spouse)*

	I'm very happy that it's over. It gets better and I've learned a lot—I've grown and hey, that's not what I was expecting to say. (Son)

One Visitor Rule

Participants from all interviews provided their perspectives on the challenge of adhering to the one designated visitor policy. Difficult for all, this policy was especially burdensome to families with young children: "At that time, one visitor was allowed that had to be the same visitor. So, our children were not allowed to visit, which was really hard on them all." (Spouse). Most designated family caregivers agreed about the guilt when absent from the unit: "I just couldn't imagine him being there all by himself—I just had to be there, it was very important to me" (Adult Child). One family caregiver remarked that not having external support (present with them in the ICU) was challenging:

> *It was absolutely harder because of COVID. I felt like I had to be there all of the time because only one person was allowed, and I didn't ever want him to feel like he was alone. He didn't understand why the kids couldn't be there—that made it very hard, not being able to have that support.* (Spouse)

Patient Advocate Role

All designated family caregivers shared their perspectives on the importance of being present that provided an opportunity for the family caregiver (who knows the patient best)

to weigh in on subtleties they may notice in the patient’s overall demeanor. Family caregivers took opportunities to be actively involved in care of their loved one:

> *I was there and I saw the breathing exercises, so then I could quiz him later on. I asked, did you do it, do you remember how long you're supposed to do it? You're supposed to do it every hour. Are you doing it? Those people out there really care, and I want you to do your breathing exercise. But what I know is that if that was just a phone call [if no visitors were allowed in the ICU], I wouldn’t know those specifics and I wouldn’t be able to watch and be an advocate.* (Spouse)

In contrast, some family caregivers described feeling distress about being involved in patient care and were waiting to be asked to weigh in. One family caregiver remarked: “Even though I was the only one there I was never asked to pipe up, to tell them about what he is really like, to advocate—I didn’t know how you know, being alone” (Spouse). The lack of invitation evoked feelings of isolation in one family caregiver: “The lesson learned is I wish I would have been more involved in rounding. I wish I would've been more invited. I know it’s hard right now, given [COVID-19] restrictions, but if I was even just listening, I would have felt included” (Adult Child).

Information Needs

Participants shared their need to receive regular and clear information from the healthcare team regarding their loved one. One family caregiver began to ask questions in order to feel more involved: “I'm a very curious person and I like to know what's going on. So, I spent a long time asking questions whenever they were there. The staff was very good at giving us answers, but yet a lot of the time we were waiting—sitting and waiting or going back and forth and waiting for them to come to your patient” (Adult Child). However, despite being present, designated family caregivers felt absent without the support of their family in the ICU. A spouse pronounced: “I was afraid to ask questions. I felt isolated. Not a lot of information was given. I felt intimidated” (Spouse). In addition, participants unanimously described the burden of having to relay medical information to remaining family members who were not allowed to visit in the ICU. The significance of other family members in the ICU was conspicuous by their absence:

> *I had to give daily updates to everybody. I would wait for a report and then I would go out to call everybody, let them know how he was doing. So many calls—I had to make myself a pretty decent schedule. The only things I had time for were to come in [to the*

ICU], make all my calls, go back to the hotel, turn on TV for a few minutes and then go to bed. (Spouse)

Emotional Distress

Designated family caregivers described feeling emotionally distressed for their critically ill loved one. One family caregiver recalled: “It was an emotional and very difficult experience. I felt scared, left out, kind of anxious” (Adult Child). Families were mindful of the unnatural and lonely feeling of being in the ICU without other family members: “I spent many hours just sitting there wondering, you know, listening to machines, beeping, very loudly. I was on an emotional rollercoaster—I couldn’t find solitude” (Spouse). Caring for a critically ill patient without support of other family, during a pandemic, sometimes involved self-affirmations:

> *I think that you fool yourself into thinking that you’re okay. I'm okay. I’m okay. I’m okay, I kept saying. You just are running on adrenaline. Right? I didn't relax until he left ICU and I know it’s the times, right. I had to tell myself that it was fine…initially anyway.* (Spouse)

Strategies for Coping with Challenges

“Mostly for my own mental health” (Adult Child), while others echoed: “I made myself a decent schedule… day in and day out. That helped for all of us” (Spouse). When hospitalized, separated, and isolated at night, one spouse recounted their strategy to cope with the challenge of restricted visitation:

> *I wrote everything down. I would write it down, what the care team said for the entire day, and then at end of each day I would write everything in a second book that I left for my husband so that he could look back and find what he needed to know, even when I wasn't there.* (Spouse)

Practicing Empathy

Participants described practicing empathy with members of the ICU care team, rather than other family caregivers, as waiting rooms were closed and caregivers from different families were not allowed to interact in the ICU. Bearing witness to the challenges faced by other family caregivers, one adult child shared: “Being able to talk to them [the ICU care team] provided a feeling of solidarity. They were going through a hard time. The talking helped, like teamwork” (Adult Child). Families recommended potential avenues for designated family caregivers to provide support to each other throughout restricted visitation: “I would suggest

a [virtual] peer group of people that have to deal with these issues, so we can exchange coping mechanisms and ideas, and show empathy for others, you know, that would be helpful" (Spouse).

Appreciation of Growth

All designated family caregivers who participated shared their perspectives of the negative impact of the pandemic and shared lessons learned from providing care:

> *I mean, once you've been through these stressful, traumatic, draining situations, you look back and reflect and think, great, I can do tough things because I've dealt with a lot.* (Spouse)

In the end, designated family caregivers, tired and isolated, described the influence of being resilient on their own, personal growth: "I'm very happy that it's over. It gets better and I've learned a lot—I've grown and hey, that's not what I was expecting to say" (Adult Child).

DISCUSSION

We conducted a semi-structured interview study to explore perspectives of family caregivers of critically ill patients on the impact of one-person designated visitor policies mandated in

ICUs during the COVID-19 pandemic. Our findings indicated that practices to control spread of the SARS-CoV-2 virus changed visitation in the ICU, which transformed the way family caregivers cared for their critically ill loved one. In the context of one-person designated visitor policies in the ICU, these changes led to complex situations that had communication and emotional consequences for family caregivers. The unintended repercussions experienced by designated family caregivers largely hinged on the notion that despite being physically present, designated family caregivers felt helpless and isolated from the ICU care team, and guilt related to being the only family member allowed to visit.

Supporting family caregivers is fundamental to the practice of critical care medicine [6] that is rarely easy [24] and has been more challenging in the COVID-19 pandemic [25]. Even with uninterrupted bedside access and idyllic support, family caregivers have high risk of long-term physical and mental health problems [26]. A one designated visitor policy at our institution that was similar to mandated policies at other Canadian [27, 28] and American [29, 30] institutions meant that if two family members were present when their loved one was admitted, they were forced to choose: who will sit alone, vigil, at the bedside, and who will walk away, leaving their critically ill family member and grieving partner behind?

The COVID-19 pandemic resulted in limitations on family caregiver engagement in the ICU and participation in care that completely reengineered their methods to cope and had potential implications on their well-being [31-33]. The issue is that public health, without an understanding of ICU care, broadly directed hospital restrictions usually without an understanding of potential adverse impact and without input and/or feedback from healthcare providers [34]. The evidence that these interventions mitigated spread of the virus (their benefit) was never measured compared to the negative impact to patients and families against which they were applied (the harm) [35-37]. The data suggests that there was harm, and that this should be considered for future pandemic planning which needs to include perspectives from family caregivers on how to best mitigate the negative effects of restricted visitation [38]. Most research has reported on short-term impacts of restricted visitation policies, few including perspectives from family caregivers themselves, and longer-term consequences of restricted visitation policies are unknown [39-41]. Added care for ICU family caregivers that emphasizes respect, dignity, and humanization, might come in the form of long-term support plans delivered by ICU family liaison teams [42-44] and psychologists [45-

47] that may help to ameliorate the impact of the COVID-19 pandemic on designated family caregivers of critically ill patients.

Prior to mandated restricted visitation policies, studies report families being offered an increasingly active role in the ICU in the participation in patient care (e.g., hygiene, orientation, mobility) [48, 49]. This is related to a growing awareness that family caregivers of ICU patients have specific needs including information to understand the diagnosis, prognosis, and treatment in the patient [50], and support in dealing with psychological distress [51]. Participation in care helps to provide families with a feeling of closeness to the patient [52] that may facilitate their sensemaking about the critical illness [53], thus alleviating their stress [54]. Performing some patient care usually left to healthcare professionals may help families to understand the caring nature of ICU treatments, which may otherwise seem highly invasive [55]. Family participation in care can also play a role in decreasing feelings of powerlessness [56] and contribute to a sense of usefulness that may help to alleviate negative mental health consequences such as guilt, grief, or burden [57]. Furthermore, a role in patient care for families may help the care team to emphasize that families are not just visitors but welcome and appreciated members in the ICU [58].

Participation in care is associated with better satisfaction among family caregivers [59]; thus, it is suggested that healthcare professionals should consider encouraging family caregivers (who wish to do so) to participate in patient care with the support of the ICU care team [52, 60, 61].

The strengths of this study include that the interview guide was informed by narratives reported in the COVID-19 pandemic [62-65], co-designed with researchers, patients, and clinicians, and tested in a pilot study with critical care nurses and intensivists. Interviews were conducted individually and at length, which allowed caregiver's time and space to share perspectives to offer important insights on the psychological burden that afflicts designated family caregivers. There are limitations to consider when interpreting the findings of our study. First, the number of participants included in this study was dependent on the interest of family caregivers in being contacted to participate in additional research projects; other studies were paused many times to conserve resources (i.e., personal protective equipment, staff) to combat the COVID-19 pandemic, which limited recruitment. We did not assess non-designated family members and it is possible that important perspectives were missed. Second, we chose a 6-month follow-up as we were cautious about grief experienced by

family caregivers who lost loved ones to critical illness [66, 67]. Third, this is a single-centre qualitative study including six family caregivers that may not be transportable to other ICU settings. Additional interviews to collect data past code saturation in order to assess meaning saturation are required for transferability of our results [68]. Finally, our small sampling frame did not achieve adequate representation of sex, gender, education, and socioeconomic status and we were not able to explore sociocultural factors, including cognitive and linguistic barriers, which might impact communication [69, 70].

It will important to discuss the fear of caregivers of having COVID vs going in Hospital, particularly before the Understanding of COVID and introduction of vaccination. Risk vs benefits

CONCLUSIONS

Designated family caregivers of critically ill patients in the ICU perceived that restricted visitation policies mandated to control COVID-19 had unintended negative repercussions. The one-person designated visitor policy meant that the designated family caregiver had to process their loves one’s prognosis and treatment, make life-changing decisions, and then relay this information to remaining family in addition to coping with their own concerns. Long-term support plans for family caregivers of critically ill patients delivered by ICU family

liaison teams and psychologists may help to ameliorate the impact of the COVID-19 pandemic on designated family caregivers of critically ill patients. Further research with larger and more diverse sample sizes are required to validate our findings from this hypothesis-generating work.

During COVID or all the time? Need to be address in discussion.

DECLARATIONS

Ethics approval and consent to participate: The Conjoint Health Research Ethics Board at the University of Calgary approved this study (Ethics ID: REB19-1000).

Consent for publication: Not applicable.

Competing interests: The authors declare that they have no competing interests.

Availability of data and materials: Data cannot be shared publicly because of patient confidentiality. Data may be available upon reasonable request from the University of Calgary research ethics board and Alberta Health Services research and innovation administration (contact via chreb@ucalgary.ca and research. administration@ahs.ca) for researchers who meet the criteria for access to confidential data.

Funding: SJM was supported by a Canadian Institutes of Health Research Doctoral Research Award. The funder had no role in study design, data collection and analysis, decision to publish, or preparation of the manuscript; no financial relationships with any organizations that might have an interest in the submitted work in the previous three years; no other relationships or activities that could have influenced the submitted work.

Authors' contributions: All designated as authors have met ICMJE criteria for authorship.

1. Substantial contributions to the conception or design of the work; or the acquisition, analysis, or interpretation of data for the work; AND
2. Drafting the work or revising it critically for important intellectual content; AND
3. Final approval of the version to be published; AND
4. Agreement to be accountable for all aspects of the work in ensuring that questions related to the accuracy or integrity of any part of the work are appropriately investigated and resolved.

All authors participated in the design and development of the protocol. SJM, KDK and KMF drafted the manuscript, and all authors read and approved the final manuscript.

Acknowledgements: The authors are grateful for the privilege of speaking with families during the ongoing pandemic. The authors would like to acknowledge the individuals who participated in developing (Bonnie Sept, Israt Yasmeen), reviewing (Melanie C. & Jillian Anglin, Chloe M. de Grood), and piloting the interview guide (Krista Wollny, Victoria Owen, Natalia Jaworska).

REFERENCES

1. Finfer S, Vincent JL: **Critical care--an all-encompassing specialty**. *N Engl J Med* 2013, **369**(7):669-670.
2. Wang S, Allen D, Kheir YN, Campbell N, Khan B: **Aging and Post-Intensive Care Syndrome: A Critical Need for Geriatric Psychiatry**. *Am J Geriatr Psychiatry* 2018, **26**(2):212-221.
3. Davidson JE, Jones C, Bienvenu OJ: **Family response to critical illness: postintensive care syndrome-family**. *Crit Care Med* 2012, **40**(2):618-624.
4. Nassar Junior AP, Besen B, Robinson CC, Falavigna M, Teixeira C, Rosa RG: **Flexible Versus Restrictive Visiting Policies in ICUs: A Systematic Review and Meta-Analysis**. *Crit Care Med* 2018, **46**(7):1175-1180.
5. Westphal GA, Moerschberger MS, Vollmann DD, Inacio AC, Machado MC, Sperotto G, Cavalcanti AB, Koenig A: **Effect of a 24-h extended visiting policy on delirium in critically ill patients**. *Intensive Care Med* 2018, **44**(6):968-970.
6. Davidson JE, Aslakson RA, Long AC, Puntillo KA, Kross EK, Hart J, Cox CE, Wunsch H, Wickline MA, Nunnally ME *et al*: **Guidelines for Family-Centered Care in the Neonatal, Pediatric, and Adult ICU**. *Crit Care Med* 2017, **45**(1):103-128.
7. **Family Visitation in the Adult Intensive Care Unit**. *Crit Care Nurse* 2017, **37**(4):88.
8. Cook D, Burns K, Finfer S, Kissoon N, Bhagwanjee S, Annane D, Sprung CL, Fowler R, Latronico N, Marshall J: **Clinical research ethics for critically ill patients: a pandemic proposal**. *Crit Care Med* 2010, **38**(4 Suppl):e138-142.
9. Subramanian S, Pamplin JC, Hravnak M, Hielsberg C, Riker R, Rincon F, Laudanski K, Adzhigirey LA, Moughrabieh MA, Winterbottom FA *et al*: **Tele-Critical Care: An Update From the Society of Critical Care Medicine Tele-ICU Committee**. *Crit Care Med* 2020, **48**(4):553-561.
10. Dhala A, Sasangohar F, Kash B, Ahmadi N, Masud F: **Rapid Implementation and Innovative Applications of a Virtual Intensive Care Unit During the COVID-19 Pandemic: Case Study**. *J Med Internet Res* 2020, **22**(9):e20143.
11. Yardley S, Rolph M: **Death and dying during the pandemic**. *BMJ* 2020, **369**:m1472.
12. Rhee J, Grant M, Detering K, Clayton J, Arthurs K: **Dying still matters in the age of COVID-19**. *Aust J Gen Pract* 2020, **49**.

13. Valley TS, Schutz A, Nagle MT, Miles LJ, Lipman K, Ketcham SW, Kent M, Hibbard CE, Harlan EA, Hauschildt K: **Changes to Visitation Policies and Communication Practices in Michigan ICUs during the COVID-19 Pandemic**. *Am J Respir Crit Care Med* 2020, **202**(6):883-885.
14. Cook DJ, Takaoka A, Hoad N, Swinton M, Clarke FJ, Rudkowski JC, Heels-Ansdell D, Boyle A, Toledo F, Dennis BB *et al*: **Clinician Perspectives on Caring for Dying Patients During the Pandemic : A Mixed-Methods Study**. *Ann Intern Med* 2021, **174**(4):493-500.
15. Munshi L, Evans G, Razak F: **The case for relaxing no-visitor policies in hospitals during the ongoing COVID-19 pandemic**. *CMAJ* 2021, **193**(4):E135-E137.
16. Kim H, Sefcik JS, Bradway C: **Characteristics of Qualitative Descriptive Studies: A Systematic Review**. *Res Nurs Health* 2017, **40**(1):23-42.
17. Tong A, Sainsbury P, Craig J: **Consolidated criteria for reporting qualitative research (COREQ): a 32-item checklist for interviews and focus groups**. *Int J Qual Health Care* 2007, **19**(6):349-357.
18. Shulman RW, Kalra S, Jiang JZ: **Validation of the Sour Seven Questionnaire for screening delirium in hospitalized seniors by informal caregivers and untrained nurses**. *BMC Geriatr* 2016, **16**:44.
19. Burns KEA, Misak C, Herridge M, Meade MO, Oczkowski S, Patient, Family Partnership Committee of the Canadian Critical Care Trials G: **Patient and Family Engagement in the ICU. Untapped Opportunities and Underrecognized Challenges**. *Am J Respir Crit Care Med* 2018, **198**(3):310-319.
20. Braun V, Clarke V: **Using thematic analysis in psychology**. *Qualitative Research in Psychology* 2006, **3**(2):77-101.
21. Fereday J, Muir-Cochrane E: **Demonstrating Rigor Using Thematic Analysis: A Hybrid Approach of Inductive and Deductive Coding and Theme Development**. *International Journal of Qualitative Methods* 2006, **5**(1):80-92.
22. Oxenboll-Collet M, Egerod I, Christensen V, Jensen J, Thomsen T: **Nurses' and physicians' perceptions of Confusion Assessment Method for the intensive care unit for delirium detection: focus group study**. *Nurs Crit Care* 2018, **23**(1):16-22.
23. Sandelowski M: **What's in a name? Qualitative description revisited**. *Res Nurs Health* 2010, **33**(1):77-84.

24. Brown SM, Azoulay E, Benoit D, Butler TP, Folcarelli P, Geller G, Rozenblum R, Sands K, Sokol-Hessner L, Talmor D *et al*: **The Practice of Respect in the ICU**. *Am J Respir Crit Care Med* 2018, **197**(11):1389-1395.

25. Andrist E, Clarke RG, Harding M: **Paved With Good Intentions: Hospital Visitation Restrictions in the Age of Coronavirus Disease 2019**. *Pediatr Crit Care Med* 2020, **21**(10):e924-e926.

26. October T, Dryden-Palmer K, Copnell B, Meert KL: **Caring for Parents After the Death of a Child**. *Pediatr Crit Care Med* 2018, **19**(8S Suppl 2):S61-S68.

27. **AHS COVID-19 PROVINCIAL GUIDANCE FOR DESIGNATED SUPPORT AND VISITOR ACCESS IN ACUTE, AMBULATORY AND EMERGENCY/URGENT CARE**. In.: Alberta Health Services; 2021.

28. **Visitor guidelines during COVID-19**. In.: Fraser Health; 2021.

29. **Update Visitor Policy for COVID-19**. In.: University of Michigan Health; 2021.

30. **General Visitation Guidelines**. In.: New York Presbyterian Hospitals; 2021.

31. Kent EE, Ornstein KA, Dionne-Odom JN: **The Family Caregiving Crisis Meets an Actual Pandemic**. *J Pain Symptom Manage* 2020, **60**(1):e66-e69.

32. Cattelan J, Castellano S, Merdji H, Audusseau J, Claude B, Feuillassier L, Cunat S, Astrie M, Aquin C, Buis G *et al*: **Psychological effects of remote-only communication among reference persons of ICU patients during COVID-19 pandemic**. *J Intensive Care* 2021, **9**(1):5.

33. Leggett A, Carmichael A, Leonard N, Robinson-Lane S, Li S, Oxford G, Wisniewski M, Gonzalez R: **Family Caregivers on the Frontline: Challenges of Providing Care to Post-ICU COVID-19 Patients**. *Innov Aging* 2020, **4**(Suppl 1):946-946.

34. Markowitz DM, Shoots-Reinhard B, Peters E, Silverstein MC, Goodwin R, Bjalkebring P: **Dehumanization During the COVID-19 Pandemic**. *Front Psychol* 2021, **12**:634543.

35. Downar J, Kekewich M: **Improving family access to dying patients during the COVID-19 pandemic**. *Lancet Respir Med* 2021, **9**(4):335-337.

36. Voo TC, Senguttuvan M, Tam CC: **Family Presence for Patients and Separated Relatives During COVID-19: Physical, Virtual, and Surrogate**. *J Bioeth Inq* 2020, **17**(4):767-772.

37. Munshi L, Evans G, Razak F: **The case for relaxing no-visitor policies in hospitals during the ongoing COVID-19 pandemic**. *Canadian Medical Association Journal* 2021, **193**(4):E135.

38. Budnick A, Hering C, Eggert S, Teubner C, Suhr R, Kuhlmey A, Gellert P: **Informal caregivers during the COVID-19 pandemic perceive additional burden: findings from an ad-hoc survey in Germany**. *BMC Health Serv Res* 2021, **21**(1):353.

39. Stall NM, Johnstone J, McGeer AJ, Dhuper M, Dunning J, Sinha SK: **Finding the Right Balance: An Evidence-Informed Guidance Document to Support the Re-Opening of Canadian Nursing Homes to Family Caregivers and Visitors during the Coronavirus Disease 2019 Pandemic**. *J Am Med Dir Assoc* 2020, **21**(10):1365-1370 e1367.

40. Tupper SM, Ward H, Parmar J: **Family Presence in Long-Term Care During the COVID-19 Pandemic: Call to Action for Policy, Practice, and Research**. *Can Geriatr J* 2020, **23**(4):335-339.

41. Zeh RD, Santry HP, Monsour C, Sumski AA, Bridges JFP, Tsung A, Pawlik TM, Cloyd JM: **Impact of visitor restriction rules on the postoperative experience of COVID-19 negative patients undergoing surgery**. *Surgery* 2020, **168**(5):770-776.

42. Belli LF: **[Recommendations for communicating bad news by phone during the SARS-CoV-2 pandemicRecomendacoes para a comunicacao de mas noticias por telefone durante a pandemia do SARS-CoV-2]**. *Recomendaciones para la comunicacion de malas noticias por telefono durante la pandemia por SARS-CoV-2* 2020, **44**:e69.

43. Griffin KM, Karas MG, Ivascu NS, Lief L: **Hospital Preparedness for COVID-19: A Practical Guide from a Critical Care Perspective**. *American Journal of Respiratory & Critical Care Medicine* 2020, **201**(11):1337-1344.

44. Janssen DJA, Ekstrom M, Currow DC, Johnson MJ, Maddocks M, Simonds AK, Tonia T, Marsaa K: **COVID-19: guidance on palliative care from a European Respiratory Society international task force**. *European Respiratory Journal* 2020, **56**(3):2002583.

45. Landa-Ramírez E, Domínguez-Vieyra NA, Hernández-Nuñez ME, Díaz-Vásquez LP, Santana-García IA: **Psychological Support During COVID-19 Death Notifications: Clinical Experiences From a Hospital in Mexico**. *Psychological Trauma: Theory, Research, Practice & Policy* 2020, **12**:518-520.

46. Lissoni B, Del Negro S, Brioschi P, Casella G, Fontana I, Bruni C, Lamiani G: **Promoting Resilience in the Acute Phase of the COVID-19 Pandemic: Psychological Interventions for Intensive Care Unit (ICU) Clinicians and Family Members**. *Psychological Trauma: Theory, Research, Practice & Policy* 2020, **12**:S105-S107.

47. Viaux S, Maurice P, Cohen D, Jouannic JM: **Giving birth under lockdown during the COVID-19 epidemic**. *Journal of Gynecology Obstetrics and Human Reproduction* 2020, **49**(6):101785.

48. Robinson SM, Mackenzie-Ross S, Campbell Hewson GL, Egleston CV, Prevost AT: **Psychological effect of witnessed resuscitation on bereaved relatives**. *Lancet* 1998, **352**(9128):614-617.

49. Robinson A, Thomson R: **Variability in patient preferences for participating in medical decision making: implication for the use of decision support tools**. *Qual Health Care* 2001, **10 Suppl 1**:i34-38.

50. Azoulay E, Chevret S, Leleu G, Pochard F, Barboteu M, Adrie C, Canoui P, Le Gall JR, Schlemmer B: **Half the families of intensive care unit patients experience inadequate communication with physicians**. *Crit Care Med* 2000, **28**(8):3044-3049.

51. Hudson P, Trauer T, Kelly B, O'Connor M, Thomas K, Summers M, Zordan R, White V: **Reducing the psychological distress of family caregivers of home-based palliative care patients: short-term effects from a randomised controlled trial**. *Psychooncology* 2013, **22**(9):1987-1993.

52. Azoulay E, Pochard F, Chevret S, Arich C, Brivet F, Brun F, Charles PE, Desmettre T, Dubois D, Galliot R *et al*: **Family participation in care to the critically ill: opinions of families and staff**. *Intensive Care Med* 2003, **29**(9):1498-1504.

53. Davidson JE, Daly BJ, Agan D, Brady NR, Higgins PA: **Facilitated sensemaking: a feasibility study for the provision of a family support program in the intensive care unit**. *Crit Care Nurs Q* 2010, **33**(2):177-189.

54. Davidson JE, Zisook S: **Implementing Family-Centered Care Through Facilitated Sensemaking**. *AACN Adv Crit Care* 2017, **28**(2):200-209.

55. Dirik A, Sandhu S, Giacco D, Barrett K, Bennison G, Collinson S, Priebe S: **Why involve families in acute mental healthcare? A collaborative conceptual review**. *BMJ Open* 2017, **7**(9):e017680.

56. Felten-Barentsz KM, van de Wetering-van Dongen VA, Vloet L, Koenders N, Nijhuis-van der Sanden MWG, Hoogeboom TJ: **Family participation during physical activity in the intensive care unit: A longitudinal qualitative study**. *Journal of Critical Care* 2021.

57. Naef R, von Felten S, Ernst J: **Factors influencing post-ICU psychological distress in family members of critically ill patients: a linear mixed-effects model**. *Biopsychosoc Med* 2021, **15**(1):4.

58. Cano A, Scaturo DJ, Sprafkin RP, Lantinga LJ, Fiese BH, Brand F: **Family Support, Self-Rated Health, and Psychological Distress**. *Prim Care Companion J Clin Psychiatry* 2003, **5**(3):111-117.
59. Wasser T, Pasquale MA, Matchett SC, Bryan Y, Pasquale M: **Establishing reliability and validity of the critical care family satisfaction survey**. *Crit Care Med* 2001, **29**(1):192-196.
60. Marsden C: **Family-centered critical care: an option or obligation?** *Am J Crit Care* 1992, **1**(3):115-117.
61. Quill TE, Brody H: **Physician recommendations and patient autonomy: finding a balance between physician power and patient choice**. *Ann Intern Med* 1996, **125**(9):763-769.
62. Benatti SV: **Love in the Time of Corona**. *Ann Intern Med* 2020, **172**(9):628.
63. Landry A, Ouchi K: **Story of human connection**. *Emerg Med J* 2020, **37**(8):526.
64. Neville TH: **COVID-19: A Time for Creative Compassion**. *J Palliat Med* 2020, **23**(7):990-991.
65. Life Lines Team c: **Restricted family visiting in intensive care during COVID-19**. *Intensive Crit Care Nurs* 2020, **60**:102896.
66. Chochinov HM, McClement S, Hack T, Thompson G, Dufault B, Harlos M: **Eliciting Personhood Within Clinical Practice: Effects on Patients, Families, and Health Care Providers**. *J Pain Symptom Manage* 2015, **49**(6):974-980 e972.
67. Choi J, Son YJ, Tate JA: **Exploring positive aspects of caregiving in family caregivers of adult ICU survivors from ICU to four months post-ICU discharge**. *Heart Lung* 2019, **48**(6):553-559.
68. Hennink MM, Kaiser BN, Marconi VC: **Code Saturation Versus Meaning Saturation: How Many Interviews Are Enough?** *Qual Health Res* 2017, **27**(4):591-608.
69. Ortega G, Rodriguez JA, Maurer LR, Witt EE, Perez N, Reich A, Bates DW: **Telemedicine, COVID-19, and disparities: Policy implications**. *Health Policy Technol* 2020, **9**(3):368-371.
70. Chua IS, Jackson V, Kamdar M: **Webside Manner during the COVID-19 Pandemic: Maintaining Human Connection during Virtual Visits**. *J Palliat Med* 2020, **23**(11):1507-1509.

FIGURE CAPTIONS

Figure 1. Flow diagram of selection of family caregivers for interviews

SUPPORTING INFORMATION

S Table 1. Consolidated criteria for reporting qualitative studies (COREQ)

S Table 2. Interview guide

Figure 1

Click here to access/download;Figure;Figure 1.png

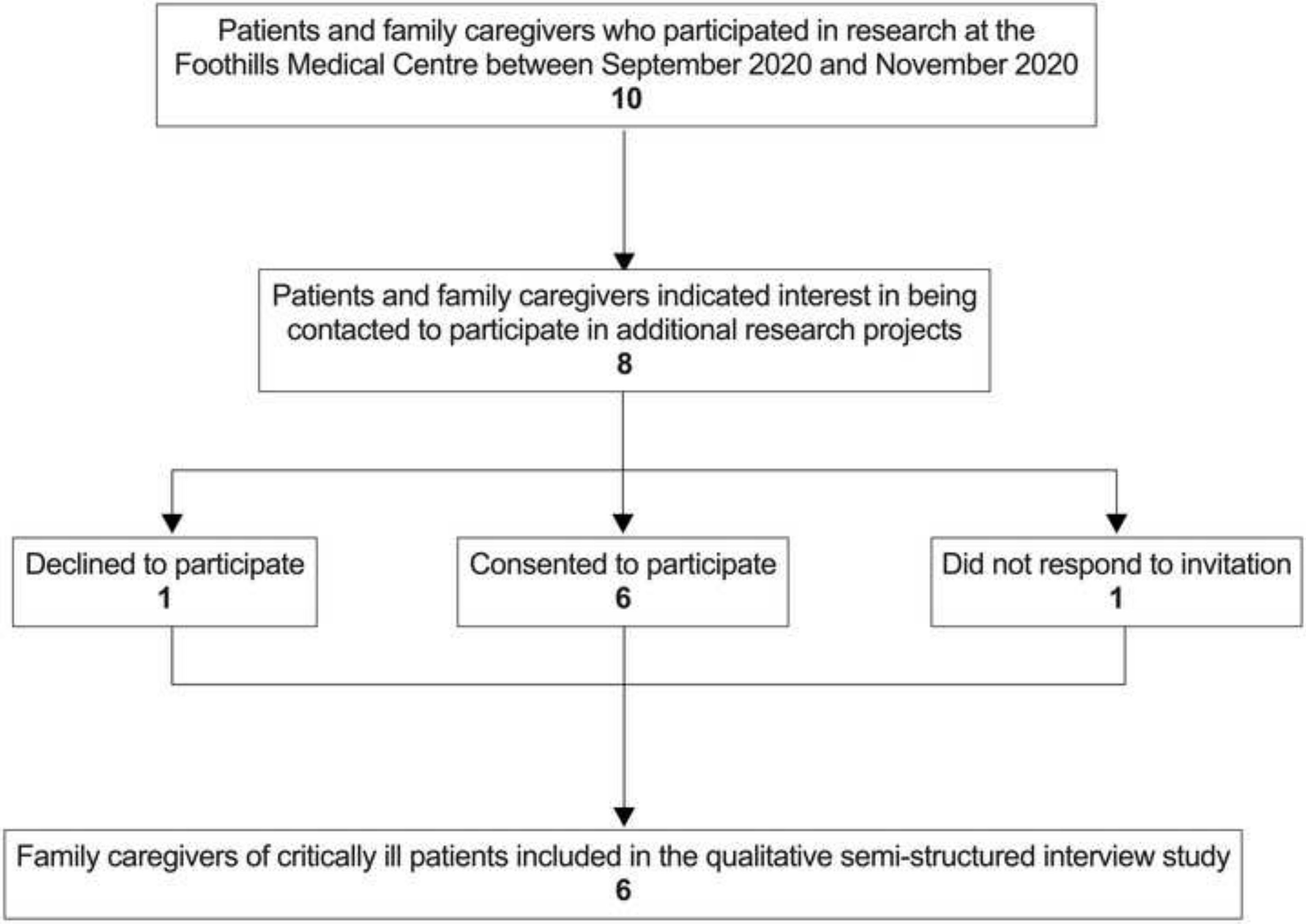

S Table 1

Click here to access/download
Supporting Information
S Table 1.docx

S Table 2

Click here to access/download
Supporting Information
S Table 2.docx
